# Pathology of Urinary Bladder in *Pearsonema* spp. Infected Wildlife from Central Italy

**DOI:** 10.3390/pathogens10040474

**Published:** 2021-04-14

**Authors:** Claudia Eleni, Alessia Mariacher, Goffredo Grifoni, Elena Cardini, Sara Tonon, Andrea Lombardo, Antonino Barone, Gianluca Fichi

**Affiliations:** 1 Istituto Zooprofilattico Sperimentale delle Regioni Lazio e Toscana, Via Appia Nuova 1411, 00178 Roma, Italy; claudia.eleni@izslt.it (C.E.); goffredo.grifoni@izslt.it (G.G.); sara.tonon-esterno@izslt.it (S.T.); 2 Istituto Zooprofilattico Sperimentale delle Regioni Lazio e Toscana, Viale Europa 30, 58100 Grosseto, Italy; elena.cardini-esterno@izslt.it (E.C.); gianluca.fichi@izslt.it (G.F.); 3 Istituto Zooprofilattico Sperimentale delle Regioni Lazio e Toscana, Via U. della Faggiola, 52100 Arezzo, Italy; andrea.lombardo@izslt.it; 4 Istituto Zooprofilattico Sperimentale delle Regioni Lazio e Toscana, Strada Terme, 01100 Viterbo, Italy; antonino.barone@izslt.it

**Keywords:** eosinophilic cystitis, mustelids, *Pearsonema*, red fox, urinary capillariosis, wolf

## Abstract

The genus *Pearsonema*, in the nematode family *Capillariidae*, includes several species that parasitize the urinary bladders of wild and domestic carnivores. The infection has been reported worldwide from several wildlife species, including canids, mustelids, and felids, but the pathological aspects have seldom been investigated. In order to assess the presence and severity of the lesions in *Pearsonema*-infected wildlife, we performed a parasitological and pathological examination of urinary bladders from 72 animals, belonging to the families *Canidae* (red fox *Vulpes vulpes*, *n* = 28, and wolf *Canis lupus*, *n* = 29) and *Mustelidae* (beech marten *Martes foina*, *n* = 3; pine marten *Martes martes*, *n* = 2; and European badger *Meles meles*, *n* = 10). A greater prevalence of infection for canids (64.91%; 95% confidence interval (95% CI), 52.52–77.30%) than for mustelids (13.33%) (*p* < 0.001) was recorded. The prevalence of infection in red foxes was 75.0% (95% CI, 58.96–91.04%), in accordance with other reports from European countries, supporting the role of this species as a reservoir for infection. Eosinophilic cystitis was observed in 34 out of the 72 examined animals (47.22%). The influence of *Pearsonema* sp. infection on the occurrence of eosinophilic cystitis was statistically significant in wolves (*p* < 0.01), which were also affected by more severe histological lesions compared to foxes.

## 1. Introduction

*Pearsonema plica*, syn. *Capillaria plica* (Rudolphi, 1819), is a cosmopolitan nematode that infects several species of canids, felids, and mustelids, and it is the agent of the so-called “urinary capillariosis.” Adult parasites are thread-like, whitish nematodes, belonging to the family *Capillariidae* (order Enoplida) that reportedly live attached to or embedded in the mucosa of the urinary bladder, while the ureters and renal pelvis are seldom affected [1]. Beyond *P. plica*, several other species have been described in the genus *Pearsonema*, and all of them are parasites of the urinary bladder in carnivores [2]. In particular, *P. feliscati* infects felids, while *P. mucronata* infects several mustelid species [3,4].

The life cycle of *Pearsonema* sp. is indirect, with the participation of an obligate intermediate host. In *P. plica* and *P. mucronata*, the intermediate host has been identified in several earthworm species belonging to the family *Lumbricidae*, such as *Lumbricus terrestris*, *L. rubellus*, and *Dendrodrilus rubidus* [4], while the life cycle of *P. feliscati* is thought to be similar to that of *P. plica* [5]. The possibility that paratenic hosts could amplify the risk of infection for the definitive hosts whose diet is not rich in earthworms has been posited [5]. In *P. plica* and *P. mucronata*, it has been observed that adult females shed their eggs in the urine of the definitive host. A first-stage larva (L1) then develops within the egg in a span of time depending on environmental conditions, and it has to be ingested by an earthworm of the family *Lumbricidae* in order to become infective for the definitive host. The infective larvae are presumably carried from the small intestine to the urinary system by the lymph–bloodstream [1,4,5].

*Pearsonema* sp. infection has been reported worldwide in the context of broader parasitological surveys in numerous wildlife species [6,7,8,9,10,11]. The red fox (*Vulpes vulpes*) is generally considered a reservoir for the transmission of *P. plica* infection to both wild and domestic animals, due to a high prevalence of infection [8,12,13,14,15,16,17], its synanthropic habits, and its increasing presence in periurban areas [18,19,20].

*Pearsonema* sp. infection in domestic animals is frequently asymptomatic, and the presence of eggs in the urine sediment is mainly regarded as an occasional finding [21,22]. Infected animals can sometimes present with severe clinical signs of the lower urinary tract [1,23], but related histopathologic findings have been rarely investigated [24,25,26]. As for wildlife species, in most instances where gross and histologic findings of the urinary bladder were evaluated, lesions were absent or consisted of mild inflammatory infiltrates [15,17,27,28]. Only in a few cases were severe or chronic cystitis described [15,29,30]. The influence of infection on the occurrence of cystitis was not deemed to be significant in a study on 112 red foxes infected by *P. plica* [15], and generally, a correlation between the presence of infection and bladder lesions in wildlife has not been clearly established. 

In the present study, a parasitological and pathological examination of urinary bladders was performed in five different carnivore species, belonging to the families *Canidae* (the red fox and wolf *Canis lupus*) and *Mustelidae* (the beech marten *Martes foina*, pine marten *Martes martes*, and European badger *Meles meles*). Our aim was to assess the presence of lesions in *Pearsonema*-infected wildlife, evaluating for the first time the occurrence of lesions in mustelids. The prevalence of infection was compared between canid and mustelid families and between canid species. The influence of *Pearsonema* sp. infection on the occurrence of eosinophilic cystitis and the severity of histological lesions were also evaluated and compared between the different species.

## 2. Results

### 2.1. Animals and Parasitology

The urinary bladders from 72 carnivores were examined, 57 belonging to the family *Canidae* (79.2%) and 15 belonging to the family *Mustelidae* (20.8%). The overall prevalence of *Pearsonema* sp. infection in carnivores, as established based on the presence of nematodes or eggs in different samples, was 54.17% (95% CI, 42.66–65.68%). The prevalence was higher for *Canidae* (64.91%; 95% CI, 52.52–77.30%) than *Mustelidae* (13.33%) (*p* < 0.001), and particularly the red fox resulted in being the most infected species (75%; 95% CI, 58.96–91.04%), followed by the wolf (55.17%; 95% CI, 37.07–73.27%). No statistical difference was observed in the prevalence between the red fox and the wolf (*p* = 0.166) (Table 1).

**Figure 1:**
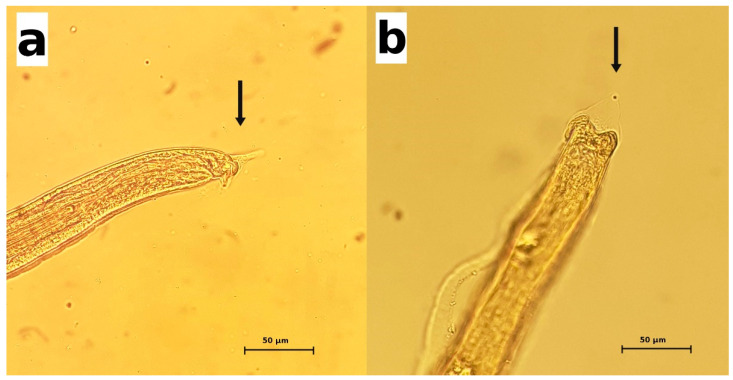
*Pearsonema plica*. The posterior end of male parasites. Arrows indicate the characteristic triangular caudal ala. (**a**) lateral view; (**b**) dorsal view.

Whenever intact parasites were isolated from the urine specimens or bladders, they were morphologically examined. The adult females were about 28–55 mm in length × 52–70 µm at their maximum width. The adult males were about 22–30 mm in length × 48–50 µm at their maximum width. The posterior end of the male parasites showed two dorsolateral lobes and a triangular terminal caudal ala (Figure 1). The males possessed a very long and thin spicule (measuring up to 4.8 mm × 7.8 µm), provided with a non-spiny spicule sheath (Figure 1). The eggs were oval and colorless, with bipolar plugs and a rough eggshell surface, and measured 59.8–67.6 µm × 33.8–36.4 µm. Based on these morphologic features, the adult parasites were identified as *Pearsonema plica*. In 13 cases, the identification at the species level was not possible because only eggs were recovered, not adult parasites.

### 2.2. Gross Pathology 

**Figure 2:**
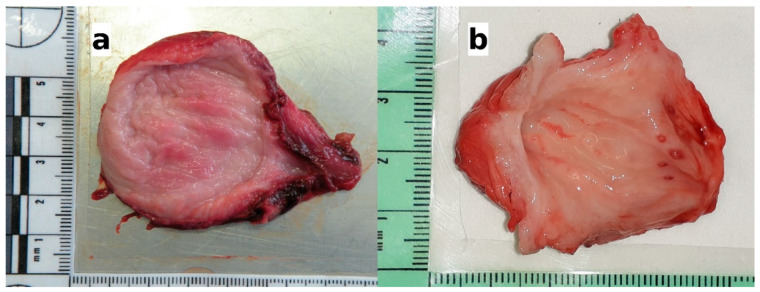
The gross lesions of the urinary bladders observed in the infected animals. (**a**) Wolf: congested areas of mucosal surface; histologically, moderate eosinophilic cystitis was observed (see Figure 3b); (**b**) wolf: congested areas in the body of the bladder and raised hemorrhagic nodules in the area around the urethral opening; histologically, hyperplasia of the lymphoid nodules, along with perinodular hemorrhages in the mucosa and severe eosinophilic cystitis were observed (see Figure 3c).

Overall, the urinary bladders were rarely affected by macroscopic lesions (*n* = 15/72; 20.83%). The observed lesions included full-thickness hemorrhages of the bladder wall (*n* = 5), congested areas of mucous membranes (*n* = 4), small whitish spots (*n* = 4), or a combination of congestion and raised hemorrhagic nodules (*n* = 2). Alterations in the thickness or texture of the bladder mucosa were not considered as macroscopic lesions, since they greatly varied with the degree of distension of the urinary bladder. The five specimens showing extensive hemorrhage, belonging to one badger and four wolves, were negative for the infection, and the reason for the alteration was consistent with traumatic events (road traffic accidents with blunt force trauma to the abdomen). The remaining 10 samples, belonging to red foxes (*n* = 5) and wolves (*n* = 5) were positive for the parasite. The gross lesions in the infected animals consisted of focal to diffuse congested areas (Figure 2a), small whitish spots 1–2 millimeters in diameter, or hemorrhagic nodules (Figure 2b). The lesions mainly affected the body of the urinary bladder; only in one positive fox did the lesion consist of focal congestion limited to the urethral opening in the trigone region. 

### 2.3. Histology

**Figure 3:**
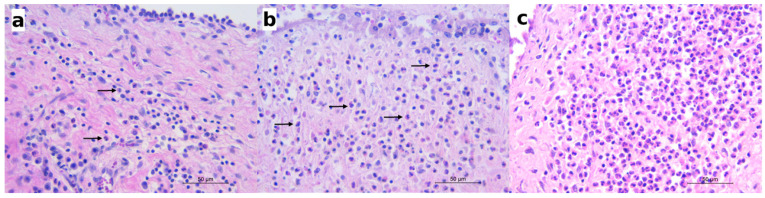
Histologically, mild to severe eosinophilic cystitis was observed in the infected animals. (**a**) Red fox: mild eosinophilic cystitis; scattered eosinophils (arrows) in the mucosal lamina propria along with lymphocytes and plasma cells; hematoxylin and eosin (HE); (**b**) wolf: moderate eosinophilic cystitis; eosinophils (arrows) arranged in small aggregates in the lamina propria; HE; (**c**) wolf: severe eosinophilic cystitis; numerous eosinophils infiltrating the mucosal lamina propria; HE.

The histological examinations revealed eosinophilic inflammation of the bladder mucosa in 34 out of 72 carnivores (47.22%). In the remaining 38 animals, no eosinophilic inflammation or other microscopic changes were detected in the bladder histological sections. The most frequently observed lesion of the bladder was a mild (“grade 1”) eosinophilic cystitis, characterized by scattered eosinophils or small aggregates of two to three eosinophils in the lamina propria of the mucosa (Figure 3a). Mild eosinophilic cystitis was observed in 26 animals belonging to all the species investigated, including 1 pine marten, 3 badgers, 1 fox, and 1 wolf, which was negative for *Pearsonema* sp. (Table 2). Moderate (“grade 2”) eosinophilic cystitis was described in five infected wolves and one infected fox, where eosinophils were arranged in small cords of three to four eosinophils or as aggregates of four or more eosinophils, distributed multifocally or diffusely (Figure 3b). In grade 2 cystitis, clusters of eosinophils were also frequently observed within the blood vessels of the submucosa. Severe (“grade 3”) cystitis was characterized by the eosinophils diffusely and densely infiltrating the lamina propria or arranged in large aggregates (Figure 3c). Severe cystitis was only observed in two infected wolves (Table 2), in which the eosinophilic infiltrate extended into the submucosa and, in some cases, into the muscular layer, involving blood vessels and the perivascular area.

**Figure 4:**
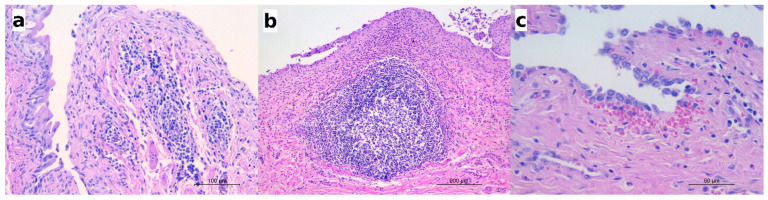
The histological features observed in the infected animals along with eosinophilic cystitis. (**a**) Red fox: numerous lymphocytes and plasma cells infiltrating the lamina propria and the submucosa also visible within the blood vessels and in the perivascular areas; HE; (**b**) wolf: the hyperplasia of a lymphoid nodule in the submucosa and the dense infiltrate of eosinophils along with a few lymphocytes and plasma cells in the lamina propria; HE; (**c**) wolf: a small hemorrhage below the mucosal epithelium; HE.

In 26 *P. plica*-infected canids (13 red foxes and 13 wolves), lymphocytic or lympho-plasmacytic infiltrates were also recorded (Figure 4a), with inflammatory cells distributed in the lamina propria, within the blood vessels of the submucosa, or as perivascular infiltrates.

In five infected wolves affected by moderate or severe eosinophilic cystitis, hyperplasia of the lymphoid nodules in the mucosa was also observed (Figure 4b), along with perinodular hemorrhages (as seen in the urinary bladder in Figure 2b), hyperaemia, and scattered microhemorrhages in the lamina propria (Figure 4c). Lymphoid nodule hyperplasia was not described in any species other than the wolf or in cases of only mild cystitis. 

A mild eosinophilic and lympho-plasmacytic infiltrate was found around the ureteral openings in two *P. plica*-infected wolves, which were affected by moderate and severe eosinophilic cystitis, respectively. 

**Figure 5:**
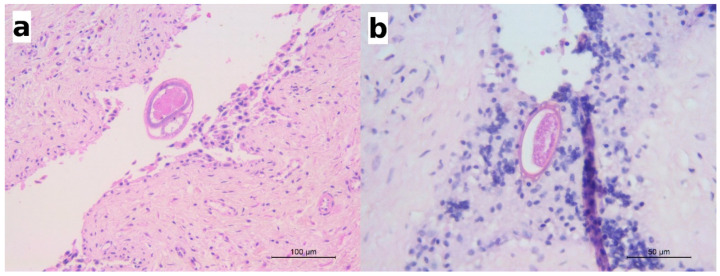
(**a**) Wolf: a section of an adult nematode in the lumen of urinary bladder; scattered eosinophils are visible in the lamina propria; HE; (**b**) European badger: a barrel-shaped egg with bipolar plugs is observed in this section; HE.

Sporadically, sections of nematodes or bipolar eggs were seen in the lumen of the urinary bladder, adjacent to the mucosal epithelium (Figure 5). The mucosal epithelium did not show significant changes but for a multifocal detachment of epithelial cells, occasionally observed in correspondence with the presence of parasite sections in the lumen (as in Figure 5a).

The absence of eosinophilic infiltrates or other microscopic changes was observed in one wolf and ten red foxes positive for *P. plica* infection. These animals were scored as a “grade 0” cystitis (Table 2).

### 2.4. Association between Pearsonema sp. and Eosinophilic Cystitis

Eosinophilic cystitis was observed in 71.74% (28/39) (Table 3) of the *Pearsonema* sp.-infected carnivores, in which the influence of the parasite on the occurrence of cystitis resulted in being statistically significant (*p* < 0.01). These results were confirmed in the *Canidae* family with 70.27% (26/36) of the *P. plica*-positive animals showing eosinophilic cystitis but not in the *Mustelidae* (*p* = 0.143), even though both the *Pearsonema* sp.-positive animals in this family showed eosinophilic cystitis (namely one European badger and one beech marten). As for the *Canidae* family, 93.75% of *P. plica*-positive wolves were affected by eosinophilic cystitis with a statistically significant association (*p* < 0.001), while this lesion was observed in only in 52.38% of infected red foxes (*p* = 0.184).

In addition, the histological score analysis confirmed a higher severity of eosinophilic cystitis (*p* < 0.01) in *P. plica*-infected wolves (1.50 ± 0.82) compared to infected red foxes (0.57 ± 0.60). No species of *Mustelidae* showed a significant association between infection and eosinophilic cystitis, and due to the low number of mustelids being positive at the parasitological examination, we did not evaluate the difference in the histopathological scores in the infected animals for these species.

## 3. Discussion

Among the species of wild carnivores examined in our study, the red foxes showed a high prevalence of *P. plica* infection (75.0%). This finding is in accordance with previous reports from several other European countries, where a prevalence higher than 70% has been observed in red foxes, for example in Germany [14], Lithuania [8], Serbia [17], and Denmark [13]. The prevalence of *P. plica* infection for red foxes in Italy previously reported by other authors ranged from 56.8% [16] to 90.5% [20]. Discrepancies in the prevalence of infection are expected between studies, due to the different geographical origins, diagnostic methods, possible seasonal differences in infection rate, and differences in the sex and age ratio in the sampled population [14].

A high prevalence of *P. plica* infection was also observed in wolves (55.17%), with no statistical difference in the prevalence between the red fox and the wolf (*p* = 0.166). This result is comparable to the 50% prevalence of infection observed in a smaller number of wolves from central Italy in a previous study [30]. Infections in wolves have been previously reported in Belarus, Spain, and Latvia with 13.5%, 7.4% and 41.4% lower prevalences, respectively [7,31,32].

The prevalence of infection observed in the present study was higher for canids than mustelids, but it is not possible to express definitive conclusions on the occurrence of infection in *Mustelidae*, since the number of examined animals was too low. Infection by *P. plica* has been previously reported with low prevalence or with single case reports in several mustelid species from Europe, including the beech marten [33,34,35], pine marten [36], European mink *Mustela lutreola* [37], and European badger [38,39].

We observed the infection by *P. plica* in a beech marten, while, in a positive badger, only the eggs but no adult parasites were recovered. Based on their location in the urinary bladder and their characteristic features (barrel-shaped with bipolar plugs), the eggs were attributed to the genus *Pearsonema*, which is the only capillariid known to infect the urinary bladder of wild carnivores [2,3]. However, since the specific identification of *Capillariidae* is only possible based on the morphology of the posterior end of male parasites [2], we could not rule out *P. mucronata*, which is the other *Pearsonema* species that is known to infect mustelids. *P. mucronata* has been reported in pine and beech martens from Ukraine [11], while reports from other European countries are lacking. 

A lower prevalence of infection in mustelids rather than canids was previously documented by other authors [11,35,36,37,38]. Considering that the ingestion of infected earthworms is the only documented route for acquiring the infection in carnivores, at least for the species *P. plica* and *P. mucronata* [4], some authors suggested that this finding apparently contradicts the known feeding habits for these hosts [35,40]. The possibility of definitive hosts acquiring the infection through ingestion of hitherto undocumented intermediate hosts or of putative paratenic hosts has been hypothesized [5,23,25,35,40]. 

Despite the demonstration of infection in numerous wildlife species, the pathologic significance of *Pearsonema* sp. has been infrequently assessed. In a study on 116 red foxes from Germany [14], 97% of the animals presenting gross alterations of the urinary bladder (i.e., reddish bladder mucosa with thickened walls) tested positive for the infection, but this observation was not corroborated by histological examinations. Other reported gross findings of the urinary bladder in infected animals included diffuse congestion and focal hemorrhages in a red fox [41], diffuse hyperemia in an arctic fox *Alopex lagopus* [29], and scattered hyperemic foci in a brown bear *Ursus arctos* [28]. In the present study, gross lesions in the infected animals were rarely observed and only in the canids. The presence of diffuse congestion and raised hemorrhagic nodules in two infected wolves was later confirmed at a histological examination as a severe eosinophilic cystitis with associated hyperplasia of the lymphoid nodules. However, we could not establish a correlation between the presence of infection and the macroscopic appearance of the bladder mucosa, and histologic examinations remain a necessary means to confirm observed pathologic changes [15].

The histological examinations in our study revealed the presence of mild to severe eosinophilic cystitis in 70.27% of *Pearsonema* sp.-infected canids. Particularly, 93.75% of the *P. plica*-positive wolves were affected by eosinophilic cystitis with a statistically significant association (*p* < 0.001). As for the foxes, eosinophilic cystitis was only observed in 52.38% of the infected subjects, and no significant influence of *P. plica* on the occurrence of cystitis in this species (*p* = 0.184) was evidenced, confirming previous results by Alić and colleagues [15]. The histological score analysis revealed a higher severity of eosinophilic cystitis (*p* < 0.01) in the *P. plica*-infected wolves (1.50 ± 0.82) compared to the infected red foxes (0.57 ± 0.60), which frequently showed mild or no bladder lesions. 

The high prevalence of infection in the red fox (75.0%) confirms the role of this species as a reservoir host for *P. plica*. Therefore, red foxes in urban and periurban areas are likely to contribute to the spread of the infection also to domestic animals, as previously hypothesized by other authors [14,20,35,42]. 

As for the animals belonging to *Mustelidae*, we observed two *Pearsonema* sp.-positive animals (one beech marten and one badger), which were both affected by mild eosinophilic cystitis. However, due to the low number of examined subjects, it is not possible to draw definitive conclusions on the significance of the infection for the species in this family. 

In six animals negative for *Pearsonema* sp. infection, namely one pine marten, three badgers, one fox, and one wolf, we observed mild eosinophilic cystitis. Nevertheless, this occurrence was too sporadic to assess any hypothesis for the development of eosinophilic cystitis in the absence of a parasitic infection, such as a low parasitic load, the occurrence of self-limiting infections, the clearance of the parasite by eosinophils, different infectious causes, or possible mechanisms of the parasite to evade the host’s immunologic response [15]. 

The present study also reports the absence of an eosinophilic infiltrate or other microscopic alterations in ten infected foxes and one wolf. This finding confirms the observations from other authors of cases of *P. plica* infection in the absence of cystitis in both wild and domestic animals [15,24,27]. The possible explanations for the absence of lesions in infected animals include a low parasitic load, the early stages of infection, or a very superficial attachment of the parasite to the mucosa, which does not directly damage the epithelium [15,24,26,27]. 

The attachment of *Pearsonema* sp. to the urinary bladder mucosa has not been univocally described. An endoscopic examination of infected dogs revealed that adult worms somehow attached to or embedded in the mucosa, while others freely floated in the lumen [1,43], but histological descriptions vary. Fernández-Aguilar and colleagues [29] observed the rostral end and body of an adult parasite embedded into the superficial mucosa in an arctic fox. Similarly, Waddell [24] in cats described the pharyngeal region of the parasites embedded in the mucosa beneath a single layer of epithelium, not penetrating the basal membrane, with the overlying cells stretched and distorted. No worms appeared to be attached to the bladder mucosa in another study on cats by Wilson-Hanson and Prescott [26]. Hamir and Rupprechtt [27] described the presence in infected raccoons (*Procyon lotor*) of narrow tunnels in the epithelium, in which cross-sections of nematodes or ova, or both, were observed. Similar tunnels were not reported by other authors, whereas polipoyd proliferations of the epithelium with multiple sections of burrowed parasites were observed in one red fox [15]. In the present study, we noticed that parasites easily detached from the sections once immersed in formalin. As a consequence, adult parasites were seldom observed adjacent to the exfoliating transitional epithelium, while parasites penetrating the mucosa or tunnel-like formations were never detected. 

As for the parasite load, we could not determine the exact number of adult worms in each infected animal, due to the fragility and thinness of capillariids, which often come in fragments or intertwined tangles. At the same time, we refrained from counting the eggs at the sediment examination, because it would not have been possible to compare the counts between the urine and bladder lavage samples. Therefore, a correlation between the intensity of infection and the presence or intensity of lesions could not be established.

## 4. Materials and Methods

### 4.1. Animals and Parasitology

Between January and December 2020, 72 wild animals belonging to the *Canidae* and *Mustelidae* families, found dead in central Italy (Latium and Tuscany regions), were necropsied. In order to assess *Pearsonema* sp. infection and the associated lesions, their urinary bladders were cut open and examined with a dissecting microscope for the presence of adult nematodes. Urine samples were collected from each bladder for a urinary sediment examination. Whenever the urine content was lacking, the bladder was pressure-washed with water. The urine and lavage fluids were centrifuged at high speed (1600 rpm) for 5 minutes, the supernatant was discarded, and a 0.1 ml droplet of the remaining sediment was then examined with bright field microscopy for the presence of eggs or larvae. The recovered nematodes were preserved in 70% ethanol until they were identified at the species level based on their morphologic and metric features [2,3].

The infected animals were identified based on the presence of adult nematodes in their urinary bladders or their urine/bladder washing samples, and/or the presence of eggs in their urine/bladder washings, and/or the presence of nematodes or eggs in their histology sections.

### 4.2. Gross Pathology and Histology

The opened urinary bladders were examined for the presence of gross lesions. Regardless of the presence of infection and/or macroscopically evident lesions, the bladder samples were fixed in 10% neutral buffered formalin, embedded in paraffin wax, sectioned at 4 μm, stained with hematoxylin and eosin (HE), and examined for histopathological lesions. 

Eosinophilic cystitis was graded as follows: 0—absent (no eosinophils); 1—mild (scattered eosinophils in the mucosal lamina propria); 2—moderate (eosinophils arranged in 1 or 2 small cords of 3–4 eosinophils or as aggregates of 4 or more eosinophils); 3—severe (diffuse infiltration of eosinophils in the mucosal propria). 

Furthermore, the presence of plasma cells or lymphocytes or the presence of prominent lymphoid nodules were recorded.

### 4.3. Data Analyses

All statistical analyses were conducted in R [44]. Fisher’s exact test was used for testing the association between the *Pearsonema* sp. and eosinophilic cystitis. An unpaired *t* test was used to evaluate the histopathological score in the different host species when possible. The differences were considered statistically significant when the *p* value was less than 0.05. The prevalence and the 95% confidence interval (95% CI) were also calculated. 

## 5. Conclusions

In the present study, eosinophilic infiltration was the most prevalent inflammatory alteration of the urinary bladder mucosa associated with *Pearsonema* sp. infection in wild carnivore species. In the *Canidae* family, *P. plica* infection influenced the occurrence of eosinophilic cystitis in wolves but not in foxes. The prevalence of infection was particularly high in red foxes, supporting the role of this species as a reservoir host. The positive wolves were affected by more severe histological grades of eosinophilic cystitis compared to the other examined species. To the best of our knowledge, this is the first description of bladder histological lesions in infected mustelids. In cases where adult forms of the parasite are not isolated from urine or urinary bladder samples, the development of molecular biology techniques would represent a fundamental aid in the diagnosis of *Pearsonema* sp. infection and identification at the species level.

## Figures and Tables

**Table 1:** The examined animals and the prevalence of *Pearsonema* sp. infection.

Species	*Pearsonema* sp. Infection		
	No. Positive/Examined	%	95% CI
**Red fox** (*Vulpes vulpes*)	21/28	75.00	58.96–91.04
**Wolf** (*Canis lupus*)	16/29	55.17	37.07–73.27
Total ***Canidae*** =	37/57	64.91	52.52–77.30
**Beech marten** (*Martes foina*)	1/3	33.33	0–86.68
**European badger** (*Meles meles*)	1/10	10.00	0–28.59
**Pine marten** (*Martes martes*)	0/2	0	0–0
Total ***Mustelidae*** =	2/15	13.33	0–30.54
Total **Carnivores** =	39/72	54.17	42.66–65.68

**Table 2:** The occurrence of eosinophilic cystitis in *Pearsonema* sp.-positive and -negative examined animals with grading of eosinophilic cystitis and associated histological findings. 0—absent; 1—mild; 2—moderate; 3—severe.

	Species	*Pearsonema* sp. Infection	Eosinophilic Cystitis				Other Histological Features		
			0	1	2	3	Lymphocytes	Plasma Cells	Lymphoid Nodules
*Canidae*	**Red fox** (*Vulpes vulpes*)	Positive	10	10	1		4	5	
		Negative	6	1					
		Total	16	11	1		4	5	
	**Wolf** (*Canis lupus*)	Positive	1	8	5	2	9	8	5
		Negative	12	1					
		Total	13	9	5	2	9	8	5
	Total *Canidae*	Positive	11	18	6	2	13	13	5
		Negative	18	2					
		Total	29	20	6	2	13	13	5
*Mustelidae*	**Beech marten** (*Martes foina*)	Positive		1					
		Negative	2						
		Total	2	1					
	**European badger** (*Meles meles*)	Positive		1			1		
		Negative	6	3			3	2	
		Total	6	4			4	2	
	**Pine marten** (*Martes martes*)	Positive							
		Negative	1	1			1	1	
		Total	1	1			1	1	
	Total *Mustelidae*	Positive		2			1		
		Negative	9	4			4	3	
		Total	9	6			5	3	
	**Total Carnivores**	Positive	11	20	6	2	14	13	5
		Negative	27	6			4	3	
		Total	38	26	6	2	18	16	5

**Table 3:** The prevalence and grading of eosinophilic cystitis in *Pearsonema* sp.-infected animals.

Species	Number of C+/P+ Animals ^1^	%	Cystitis Score (Mean ± SD)
**Red fox** (*Vulpes vulpes*)	11/21	52.38	0.57 ± 0.60
**Wolf** (*Canis lupus*)	15/16	93.75	1.50 ± 0.82
Total ***Canidae*** =	26/37	70.27	0.97 ± 0.83
**Beech marten** (*Martes foina*)	1/1	100.00	1.0 ± 0.00
**European badger** (*Meles meles*)	1/1	100.00	1.0 ± 0.00
**Pine marten** (*Martes martes*)	0	0.00	N.A. ^2^
Total ***Mustelidae*** =	2/2	100.00	1.0 ± 0.00
Total **Carnivores** =	28/39	71.74	0.93 ± 0.80

^1^ C+—cystitis positive animals; P+—*Pearsonema* sp.-positive animals; ^2^ N.A.—not available.

## Data Availability

Not applicable.

## References

[1] Basso W., Spänhauer Z., Arnold S., Deplazes P. (2014). *Capillaria plica* (syn. *Pearsonema plica*) infection in a dog with chronic pollakiuria: Challenges in the diagnosis and treatment. Parasitol. Int..

[2] Moravec F. (1982). Proposal of a new systematic arrangement of nematodes of the family Capillariidae. Folia Parasitol..

[3] Beverley-Burton M. (1980). The taxonomy of *Capillaria* spp. (Nematoda: Trichuroidea) in carnivorous mammals from Ontario, Canada. Syst. Parasitol..

[4] Moravec F., Prokopic J., Shlikas A.V. (1987). The biology of nematodes of the family Capillariidae Neveu-Lemaire, 1936. Folia Parasitol..

[5] Bowman D.D., Hendrix C.M., Lindsay D.S., Barr S.C., Bowman D.D. (2002). Feline Clinical Parasitology.

[6] Krone O., Guminsky O., Meinig H., Herrmann M., Trinzen M., Wibbelt G. (2008). Endoparasite spectrum of wild cats (*Felis silvestris* Schreber, 1777) and domestic cats (*Felis catus* L.) from the Eifel, Pfalz region and Saarland, Germany. Eur. J. Wildl. Res..

[7] Bagrade G., Kirjuina M., Vismanis K., Ozoli J. (2009). Helminth parasites of the wolf *Canis lupus* from Latvia. J. Helminthol..

[8] Bružinskaite-Schmidhalter R., Sarkunas M., Malakauskas A., Mathis A., Torgerson P.R., Deplazes P. (2012). Helminths of red foxes (*Vulpes vulpes*) and raccoon dogs (*Nyctereutes procyonoides*) in Lithuania. Parasitology.

[9] Franssen F., Nijsse R., Mulder J., Cremers H., Dam C., Takumi K., Van Der Giessen J. (2014). Increase in number of helminth species from Dutch red foxes over a 35-year period. Parasites Vectors.

[10] Takács A., Szabó L., Juhász L., Takács A., Lanszki J., Takács P., Heltai M. (2014). Data on the parasitological status of golden jackal (*Canis aureus* L., 1758) in Hungary. Acta Vet. Hung..

[11] Varodi E.I., Malega A.M., Kuzmin Y.I., Kornyushin V.V. (2017). Helminths of Wild Predatory Mammals of Ukraine. Nematodes. Vestn. Zool..

[12] Davidson R.K., Gjerde B., Vikøren T., Lillehaug A., Handeland K. (2006). Prevalence of *Trichinella* larvae and extra-intestinal nematodes in Norwegian red foxes (*Vulpes vulpes*). Vet. Parasitol..

[13] Saeed I., Maddox-Hyttel C., Monrad J., Kapel C.M.O. (2006). Helminths of red foxes (*Vulpes vulpes*) in Denmark. Vet. Parasitol..

[14] Bork-Mimm S., Rinder H. (2011). High prevalence of *Capillaria plica* infections in red foxes (*Vulpes vulpes*) in Southern Germany. Parasitol. Res..

[15] Alić A., Hodžić A., Kadrić M., Beširović H., Prašović S. (2015). *Pearsonema plica* (*Capillaria plica*) infection and associated urinary bladder pathology in red foxes (*Vulpes vulpes*) from Bosnia and Herzegovina. Parasitol. Res..

[16] Magi M., Guardone L., Prati M.C., Mignone W., Macchioni F. (2015). Extraintestinal nematodes of the red fox *Vulpes vulpes* in north-west Italy. J. Helminthol..

[17] Aleksić J., Stepanović P., Dimitrijević S., Gajić B., Bogunović D., Davidov I., Aleksić-Agelidis A., Ilić T. (2020). *Capillaria plica* in Red Foxes (*Vulpes vulpes*) from Serbia: Epidemiology and Diagnostic Approaches to Urinary Capillariosis in Domestic Carnivores. Acta Parasitol..

[18] Scott D.M., Berg M.J., Tolhurst B.A., Chauvenet A.L.M., Smith G.C., Neaves K., Lochhead J., Baker P.J. (2014). Changes in the distribution of red foxes (*Vulpes vulpes*) in urban areas in Great Britain: Findings and limitations of a media-driven nationwide survey. PLoS ONE.

[19] Mackenstedt U., Jenkins D., Romig T. (2015). The role of wildlife in the transmission of parasitic zoonoses in peri-urban and urban areas. Int. J. Parasitol. Parasites Wildl..

[20] Pelligra S., Guardone L., Riggio F., Parisi F., Maestrini M., Mariacher A., Perrucci S. (2020). *Pearsonema* spp. (Family Capillariidae, Order Enoplida) Infection in Domestic Carnivores in Central–Northern Italy and in a Red Fox Population from Central Italy. Animals.

[21] Bédard C., Desnoyers M., Lavallée M.C., Poirier D. (2002). *Capillaria* in the bladder of an adult cat. Can. Vet. J..

[22] Mariacher A., Millanta F., Guidi G., Perrucci S. (2016). Urinary capillariosis in six dogs from Italy. Open Vet. J..

[23] Rossi M., Messina N., Ariti G., Riggio F., Perrucci S. (2011). Symptomatic *Capillaria plica* infection in a young European cat. J. Feline Med. Surg..

[24] Waddell A.H. (1968). Further observations on *Capillaria feliscati* Infections in the cat. Aust. Vet. J..

[25] Senior D.F., Solomon G.B., Goldschmidt M.H., Joyce T., Bovee K.C. (1980). *Capillaria plica* infection in dogs. J. Am. Vet. Med. Assoc..

[26] Wilson-Hanson S., Prescott C.W. (1982). *Capillaria* in the bladder of the domestic cat. Aust. Vet. J..

[27] Hamir A.N., Rupprechtt C.E. (1998). A Retrospective Histopathological Survey of Capillariasis in Raccoons from the Eastern United States. J. Parasitol..

[28] Mariacher A., Eleni C., Fico R., Perrucci S. (2018). Urinary capillariosis in a free-ranging Marsican brown bear (*Ursus arctos marsicanus*). Int. J. Parasitol. Parasites Wildl..

[29] Fernández-Aguilar X., Mattsson R., Meijer T., Osterman-Lind E., Gavier-Widén D. (2010). *Pearsonema* (syn *Capillaria*) *plica* associated cystitis in a Fennoscandian arctic fox (*Vulpes lagopus*): A case report. Acta Vet. Scand..

[30] Mariacher A., Eleni C., Fico R., Ciarrocca E., Perrucci S. (2015). Research Note. *Pearsonema plica* and *Eucoleus böhmi* infections and associated lesions in wolves (*Canis lupus*) from Italy. Helminthologia.

[31] Shimalov V.V., Penkevich V.A. (2012). Helminth Fauna of the wolf (*Canis lupus* linnaeus, 1758) in Belorussian Polesie. Parazitologiya.

[32] Segovia J.M., Torres J., Miquel J., Llaneza L., Feliu C. (2001). Helminths in the wolf, *Canis lupus*, from north-western Spain. J. Helminthol..

[33] Ribas A., Milazzo C., Foronda P., Casanova J.C. (2004). New data on helminths of stone marten, *Martes foina* (Carnivora, Mustelidae), in Italy. Helminthologia.

[34] Visser M., Messner C., Rehbein S. (2011). Massive infestation with fur mites (*Lynxacarus mustelae*) of a stone marten (*Martes foina*) from Tyrol. Wien. Klin. Wochenschr..

[35] Petersen H.H., Nielsen S.T., Larsen G., Holm E., Chriél M. (2018). Prevalence of *Capillaria plica* in Danish wild carnivores. Int. J. Parasitol. Parasites Wildl..

[36] Segovia J.M., Torres J., Miquel J., Sospedra E., Guerrero R., Feliu C. (2007). Analysis of helminth communities of the pine marten, *Martes martes*, in Spain: Mainland and insular data. Acta Parasitol..

[37] Torres J., Miquel J., Fournier P., Fournier-Chambrillon C., Liberge M., Fons R., Feliu C. (2008). Helminth communities of the autochthonous mustelids *Mustela lutreola* and *M. putorius* and the introduced *Mustela vison* in south-western France. J. Helminthol..

[38] Torres J., Miquel J., Motjé M. (2001). Helminth parasites of the eurasian badger (*Meles meles* L.) in Spain: A biogeographic approach. Parasitol. Res..

[39] Takács A., Szemethy L., Takács A.A., Takács T.P., Heltai M. (2012). Data on the parasitological state of the Eurasian badger (*Meles meles*) in Hungary. Magy. Allatorv. Lapja.

[40] Seville R.S., Addison E.M. (1995). Nongastrointestinal helminths in marten (*Martes americana*) from Ontario, Canada. J. Wildl. Dis..

[41] Davidson W.R., Appel M.J., Doster G.L., Baker O.E., Brown J.F. (1992). Diseases and parasites of red foxes, gray foxes, and coyotes from commercial sources selling to fox-chasing enclosures. J. Wildl. Dis..

[42] Sréter T., Széll Z., Marucci G., Pozio E., Varga I. (2003). Extraintestinal nematode infections of red foxes (*Vulpes vulpes*) in Hungary. Vet. Parasitol..

[43] Del-Angel-Caraza J., Quijano-Hernández I.A., Soriano-Vargas E., Barbosa-Mireles M.A., Martínez-Castañeda J.S. (2018). Urinary bladder worm (*Pearsonema* sp.) infection in domestic dogs and cats in Mexico at a high altitude. Parasitol. Res..

[44] Core Team (2017). R: A Language and Environment for Statistical Computing. R Foundation for Statistical Computing, Vienna, Austria. https://www.r-project.org/.

